# Editorial: Sleep problems: diagnosis, biomarkers, interventions, and treatments

**DOI:** 10.3389/fpsyt.2023.1291522

**Published:** 2023-09-26

**Authors:** Haitham Jahrami

**Affiliations:** ^1^Government Hospitals, Manama, Bahrain; ^2^Department of Psychiatry, College of Medicine and Medical Sciences, Arabian Gulf University, Manama, Bahrain

**Keywords:** diagnosis, biomarkers, interventions, treatments, personalized and precision medicine (PPM)

Sleep problems (SP) are highly prevalent among all age groups (1). Chronic SP are linked to increased risks of obesity, cardiovascular disease, diabetes, anxiety, depression, and impaired cognition. As a global issue that greatly impacts public health, SP warrants ongoing research and clinical attention (2). Accurately diagnosing specific sleep disorders via biomarkers could enable better-targeted, personalized medicine interventions and treatments. The current Research Topic represents a collection of papers investigating the complex physiological underpinnings of sleep to better diagnose and manage SP. In the current Research Topic, fifteen original articles, five review articles, and one research protocol article were published.

Two papers reported SP among university students. Zhang L. et al. showed that SP are also prevalent among university students. Their work aimed to create a prediction model (nomogram) that could be used to detect SP. Some intervention or preventive techniques, including quitting smoking and drinking, eating a healthy diet, avoiding midday naps, attending to chronic diseases, and managing worry and stress, may be very useful in improving sleep in university students. In the original article by Guo et al., the researchers used 24-h heart rate variability (HRV) as a biomarker to investigate the sleep quality and depression symptoms of medical students. The authors reported that SP were common among medical students and were associated with depressive symptoms. Students with SP had lower SDNN during the awake period and bedtime period and lower LF in the awake period. The use of HRV analysis may provide valuable information about SP and the different stages of sleep. During the different sleep stages, HRV patterns change, reflecting the varying activities of the autonomic nervous system.

Recent umbrella reviews and meta-analyses of self-reported psychological and behavioral symptoms (PBS) in medical students showed that a global analysis of all self-reported PBS combined yielded a pooled prevalence rate of 30%. The highest reported prevalence was for SP, affecting about 42%. Appropriately targeted assessment (perhaps using HRV) and intervention efforts are clearly warranted to decrease the SP of university students during their education (3).

Three papers focused on psychiatric populations. The meta-analysis by Hu et al. showed that persistent SP for seven more days worsened symptoms scores in patients with an existing major depressive disorder. This review suggests that persistent SP lasting over a week would likely worsen symptoms or outcomes related to those health domains compared to more acute or transient sleep issues. The review by Fekih-Romdhane et al. bolstered the presence of disturbed sleep in young individuals at ultra-high risk (UHR) for psychosis, as demonstrated by subjective and objective sleep measurements such as polysomnography, sleep electroencephalograms, and actigraphy. The review discussed the potential mechanisms and processes underlying the association between sleep and psychosis and highlighted its complicated and multifaceted nature, which remains to be determined and understood. The review by Albanese et al. showed that among the various treatments proposed for treating nightmares in PTSD patients, those involving imagery rescripting appear to be the most effective. These techniques provide direct access to the traumatic contents and emotions of nightmares without overwhelming patients, and they enable healthcare workers to quickly detect and modify trauma-related negative beliefs.

Three papers focused on the diagnosis and management of SP in patients undergoing maintenance hemodialysis therapy (MHT). Xu et al. showed that in patients requiring MHT, older age, lower albumin, and calcium levels are all risk factors for SP. Lower albumin and calcium levels in these patients also correlating with SP is logically plausible, as nutritional deficiencies can affect sleep regulation. Albumin and calcium have important biological functions. In the same line of research, Tong et al. showed that SP were associated with declining renal function (the highest Cys-C, Q4) in postmenopausal women. The findings suggested that postmenopausal women should prioritize maintaining excellent sleep quality, which provides clinical evidence for the feasible early diagnosis and effective prevention of kidney disease progression, such as MHT, in postmenopausal women. Mohamed et al. highlight that collaboration between the family and healthcare workers are essential to improving the quality of sleep in patients undergoing MHT.

As a common and formal sleep disorder, OSA yielded three papers. Sun et al. found that using the slow-4 frequency range in OSA may be more specific. These findings imply that severe OSA patients exhibit frequency-related aberrant spontaneous brain activity, which could help us understand the pathology of OSA-related disorders and give us possible therapeutic targets for OSA patients. The study by Su et al. documented that despite considerable available OSA knowledge, medical residents have low confidence in OSA management. The authors suggested practical and small group teaching approaches that have the advantages of flexible time, short duration, and high participation, especially during clinical rotations. In a pediatric population, Lee et al. reported that a reduction in the overall obstructive sleep apnea (OSA) questionnaire score and the very low frequency (VLF) power of HRV mediated the improvement in the obstructive apnea-hypopnea index after adenotonsillectomy. These preliminary findings are innovative, and they point the way forward for further study into the impact of HRV-guided therapies on childhood OSA.

The study by Duong-Quy et al. highlighted that during COVID-19 SP, depression symptoms and fatigue in healthcare workers worsened among frontline healthcare workers. Earlier studies established that the COVID-19 pandemic has put great strain on the global healthcare system, necessitating the development of numerous field hospitals and isolation camps around the world. This temporary solution has resulted in the suspension of proper environmental conditions for patients and healthcare workers (4–6).

Belmon et al. suggested combining intervention mapping (IM) with the Health in All Policies (HiAP) for health promotion, which resulted in a comprehensive, evidence-based design for the implementation of a multi-sector integrated program to increase children's sleep health. Their blueprint document suggested that it be used to help build local (sleep) health promotion programs in other regions with distinct local governmental systems and cultures while keeping the policy environment in mind.

Niu et al. demonstrated high sensitivity and specificity and excellent diagnostic ability of the Pittsburgh Sleep Quality Index (PSQI), Insomnia Severity Index (ISI), and Athens Insomnia Scale (AIS) questionnaires in screening for insomnia in stroke patients. The authors concluded that each of the three questionnaires has advantages and disadvantages when assessing insomnia.

Xiang et al. aimed to investigate the changes in features and related aspects of several GABAergic system indexes in the peripheral blood of individuals with insomnia. They found that serum GABA inhibitory activity may be altered in insomnia patients, and decreased expression levels of GABA_A_ receptor α1 and α2 subunit mRNA may become a reliable indication of insomnia disorders.

While great strides have been made in understanding and treating SP, it remains an undertreated public health issue. A holistic, patient-centered approach combining multiple modalities tailored to the individual holds the most promise for optimal management of these conditions. Continued research to elucidate sleep-wake neurobiology and innovative technological solutions can help improve diagnosis, treatments, and quality of life for the millions affected by SP worldwide.

## Author contributions

HJ: Conceptualization, Data curation, Investigation, Project administration, Resources, Writing—original draft, Writing—review and editing.
